# shRNA Off-Target Effects *In Vivo*: Impaired Endogenous siRNA Expression and Spermatogenic Defects

**DOI:** 10.1371/journal.pone.0118549

**Published:** 2015-03-19

**Authors:** Hye-Won Song, Anilkumar Bettegowda, Daniel Oliver, Wei Yan, Mimi H. Phan, Dirk G. de Rooij, Mark A. Corbett, Miles F. Wilkinson

**Affiliations:** 1 Department of Reproductive Medicine, School of Medicine, University of California San Diego, La Jolla, California, United States of America; 2 Department of Physiology and Cell Biology, University of Nevada School of Medicine, Reno, Nevada, United States of America; 3 Center for Reproductive Medicine, Academic Medical Center, University of Amsterdam, Amsterdam, The Netherlands; 4 School of Pediatrics and Reproductive Health, The University of Adelaide, Adelaide, Australia; Cardiff University, UNITED KINGDOM

## Abstract

RNA interference (RNAi) is widely used to determine the function of genes. We chose this approach to assess the collective function of the highly related reproductive homeobox 3 (*Rhox3*) gene paralogs. Using a *Rhox3* short hairpin (sh) RNA with 100% complementarity to all 8 *Rhox3* paralogs, expressed from a CRE-regulated transgene, we successfully knocked down *Rhox3* expression in male germ cells *in vivo*. These *Rhox3*-shRNA transgenic mice had dramatic defects in spermatogenesis, primarily in spermatocytes and round spermatids. To determine whether this phenotype was caused by reduced *Rhox3* expression, we generated mice expressing the *Rhox3*-shRNA but lacking the intended target of the shRNA—*Rhox3*. These double-mutant mice had a phenotype indistinguishable from *Rhox3*-shRNA-expressing mice that was different from mice lacking the *Rhox3* paralogs, indicating that the *Rhox3* shRNA disrupts spermatogenesis independently of *Rhox3*. *Rhox3*-shRNA transgenic mice displayed few alterations in the expression of protein-coding genes, but instead exhibited reduced levels of all endogenous siRNAs we tested. This supported a model in which the *Rhox3* shRNA causes spermatogenic defects by sequestering one or more components of the endogenous small RNA biogenesis machinery. Our study serves as a warning for those using shRNA approaches to investigate gene functions *in vivo*.

## Introduction

RNA interference (RNAi) is a widely used approach to silence genes and thereby deduce their function. While primarily used for *in vitro* studies, RNAi has also been instrumental in deciphering the functions of many genes *in vivo*. A particularly useful application is to elucidate the functions of gene paralog families. As long as the family members are sufficiently related, a single siRNA can simultaneously knockdown all family members and thereby unveil the redundant function of the gene paralog family [1–3].

We elected to take advantage of this approach to conditionally knockdown highly related gene paralogs in the reproductive homeobox (*Rhox*) gene cluster. This gene cluster harbors 33 homeobox genes that are selectively expressed in the male and female reproductive tract, suggesting that they encode transcription factors critical for reproduction [4]. Although all *Rhox* genes are expressed in reproductive tract, their spatial and temporal expression patterns are different. For example, RHOX10 is expressed specifically in spermatogonia and early spermatocytes [5] and RHOX13 is expressed in differentiating spermatogonia and early spermatocytes [6]. RHOX5 is expressed in Sertoli cells, where it functions to promote the survival and motility of the adjacent germ cells [4,7,8]. The human *RHOX* genes are expressed specifically in germ cells in the testes and are aberrantly methylated in infertile patients [9,10].

The subject of this report is the *Rhox3* gene paralogs, which comprise eight gene copies in mice that are highly homologous with each other—displaying 98.2–99.7% sequence identity [11–14]. Using a variety of approaches, we previously reported that all the *Rhox3* gene paralogs are expressed in the adult testes except *Rhox3d* (previously called *Rhox3c*), which is likely to be a pseudogene since it harbors a premature translation termination codon [11]. The *Rhox3* gene paralogs appear to have expanded as a result of selection pressure exerted specifically in the mice lineage, as rats have only a single *Rhox3* gene [14]. While it is not known why this gene expansion occurred in the mice lineage, synonymous-to-non-synonymous ratio analysis suggests that the mouse *Rhox3* paralogs have undergone purifying selection in the amino-terminal region and weak positive selection for changes in the homeodomain region [11,12,14]. The latter suggests that the RHOX3 homeodomain has been under selection pressure to diversify, analogous to what we previously reported for the RHOX5 homeodomain region [15].

Because these eight *Rhox3* paralogs are interspersed with other *Rhox* genes, a knockout strategy to determine their collective *in vivo* function is not feasible and thus we chose to use a RNAi approach instead. To provide specificity, we used a conditional RNAi approach in which the *Rhox3* shRNA was selectively expressed in male germ cells, the cell type that we found normally express *Rhox3*. As described herein, this germ cell-specific shRNA approach successfully knocked down *Rhox3* expression in the testis *in vivo* and it led to dramatic defects in spermatogenesis.

*In vitro* RNAi studies to determine gene function in cultured cells typically incorporate controls in their experiments to distinguish whether the phenotypic defects observed are the result of depletion of the target gene product or an off-target effect [16]. In contrast, RNAi-based studies conducted *in vivo* rarely have such controls [17]. To reduce the risk of off-targeting effects in *in vivo* studies, conditional/inducible shRNA expression methods have been developed that restrict the expression of the siRNA to specific cell types, developmental stages, and/or temporal windows [18,19]. While useful, such approaches do not eliminate the possibility of off-target effects. In the study herein, we elected to address this issue by asking whether loss of the intended target of the shRNA we generated—the *Rhox3* gene paralogs—reversed the phenotypic defects caused by the *Rhox3* shRNA. An off-target effect seemed a distinct possibility given that we found that mice lacking the *Rhox3* paralogs exhibited different defects from mice expressing *Rhox3*-shRNA. Indeed, we found that elimination of the intended target did not measurably reduce or change the defects caused by the *Rhox3* shRNA, thereby demonstrating that the *Rhox3* shRNA acts by a mechanism independent of its ability to knockdown *Rhox3*. We considered and tested different mechanisms by which the *Rhox3* shRNA might cause off-target effects. This led to the discovery that *Rhox3* shRNA mice have reduced production of endogenous (endo)-small interfering (si) RNAs. In contrast to microRNAs (miRNAs), which are expressed in most of mammalian cells, endo-siRNAs are only known to be expressed in germ cells and embryonic stem cells in mammals [20–23]. Our results suggest that shRNAs should be used cautiously to elucidate the functions of genes, particularly in specialized cells such as germ cells.

## Materials and Methods

### Animals

This study was carried out in strict accordance with the Guidelines of the Institutional Animal Care and Use Committee (IACUC) at the University of California, San Diego. The protocol was approved by the IACUC at the University of California, San Diego (Permit Number: S09160). All animals were housed under a 12 h light: 12 h darkness cycle and provided with food and water *ad libitum*. Euthanasia was performed by CO2 inhalation for 5 min followed by cervical dislocation. All efforts were made to minimize suffering.

### Plasmids, cell culture, and transfection

The siDESIGN algorithm (Dharmacon Inc., Lafayette, CO) was used to design siRNA target sequences that have 100% complementarity with all 8 *Rhox3* paralogs. U6 promoter driven-shRNA expression vectors (G-1256 and G-1257) targeting *Rhox3* were generated by subcloning PCR products that contained both the target sequence and the mouse U6 promoter into the pGEM-T Easy vector, as previously described [24]. The *Rhox3* target sequences used were 5’-GGAGCAGATTCCTGAGCAT-3’ (G-1256) and 5’-GCATGTTGAAGGAGAGAGT-3’ (G-1257). The construction of the shRNA expression vector to target firefly luciferase (G-472) is previously described [25]. To generate the U6-ploxPneo *Rhox3* shRNA transgenic construct (G-1029), oligos MDA-4212 and-4213, which contained shRNA sequences, were annealed and ligated into the ApaI and EcoRI sites of the pBS/U6-ploxPneo plasmid (generously gifted by Dr. Chuxia Deng, NIH, Bethesda), as described elsewhere [26].

HeLa cells (ATCC) were maintained in Dulbecco’s modified Eagle’s medium (DMEM), supplemented with 10% fetal bovine serum and 50 mg/ml of penicillin and streptomycin and transfected with Lipofectamine-2000 following the manufacturer’s instructions. All cell culture reagents were purchased from Invitrogen Technologies (Carlsbad, CA). Cells were plated on 6-well culture plates and cotransfected with 500 ng of an expression vector encoding FLAG-tagged RHOX3 and various concentrations of U6-driven *Rhox3* shRNA plasmids. A U6-driven Luciferase shRNA plasmid was used as a control in transfection experiments. To delete the loxP-flanked neomycin cassette from the U6-ploxPneo *Rhox3* shRNA transgenic construct, a Cre recombinase (pMC-Cre) expression plasmid was co-transfected. Total cellular protein extracts were prepared 48 h after transfection.

### Transgenic mice and knock-out (KO) mice

Transgenic mice were generated by pronuclear microinjection of FVB/NJ mouse embryos (Jackson laboratory, Bar Harbor, ME) at the M. D. Anderson Cancer Center transgenic core facility. To generate U6-ploxPneo *Rhox3* shRNA mice, a 2.7-kb DNA fragment containing the U6-ploxp-neo *Rhox3* shRNA transgene in the G-1029 plasmid was excised using the restriction enzymes, AflIII and Asp718, and gel purified for microinjection. Positive transgenic mice were detected by PCR using tail DNA as a template and the primers MDA-4290 and-4291, which are specific for the U6 promoter and neomycin cassette. Four founder lines (lines 2, 11, 12, and 13) containing the transgene were obtained. All lines were mated with Stra8-iCre mice to generate double transgenic lines (expressing the *Rhox3* shRNA and CRE) to make germ cell-specific shRNA mice (*Rhox3*-shRNA mice) and the single transgenic littermates (either *Rhox3*-shRNA or Stra8-iCre) served as control (Control mice). Line 2 was subjected to detailed histological analysis and also bred with *Rhox*-c-KO mice.

To generate *Rhox*-c-floxed mice, two targeting vectors were used to insert loxP sites at the beginning (upstream genomic region of *Rhox1*) and the end (the second intron of *Rhox13*) of the *Rhox* cluster. The targeting vectors were constructed from BAC clones (Vega BioLab LLC. Philadelphia, PA). The two targeting vectors were electroporated sequentially into ES cells; in each case, cell clones that underwent homologous recombination were selected by Southern blot analysis. An ES cell clone containing both loxP sites was selected for blastocyst injection to obtain chimeric mice. Germline-transmitted *Rhox*-c-floxed mice were bred with *EIIa*-Cre mice to generate mice lacking the entire *Rhox* cluster (*Rhox*-c-KO or *Rhox*-c-heterozygote mice). The *Rhox*-c-heterozygote female mice were bred with *Rhox3*-shRNA transgenic mice to generate *Rhox*-c heterozygote; *Rhox3*-shRNA female mice and these mice were further bred with Stra8-iCre mice to obtain *Rhox*-c KO;*Rhox3*-shRNA;Stra8-iCre male mice (*Rhox*-c-KO/*Rhox3*-shRNA mice).

### RNA and protein analysis

For quantitative RT-PCR (qRT-PCR) analysis, total cellular RNA was extracted from cell lines and tissues by the Trizol method (Invitrogen, Carlsbad, CA) and converted to cDNA using iScript reverse transcription (Bio-Rad, Hercules, CA). Relative gene expression was determined by SYBR green incorporation in an Applied Biosystem StepOnePlus real-time PCR system, as described previously [4], with primers as described in Table 1. miRNA levels were determined by Taqman assay, following the manufacturer’s instructions. Endo-siRNA levels were determined as previously described [20].

**Table 1:** Primers used for qRT-PCR Analysis.

Gene	Sense	Antisense
*Rhox3*	5’-GAGAGAGTGACCAGGCTGA-3’	5’-CATTCACACCCATCCATCG-3’
*Rpl19*	5’-CTGAAGGTCAAAGGGAATGTG-3’	5’-GGACAGAGTCTTGATGATCTC-3’
*Rhox1*	5’-TTCCAGCGCACTCAGTACAT-3’	5’-CTCCTTTGAAACCAATTCTGC-3’
*Rhox2*	5’-GGTGCCCGAATTCCGC-3’	5’-ATTTCTCTCTCCTCTTCAA-3’
*Rhox4*	5’-TGTGAGTGAAGCCAGAGTT-3’	5’-AACATGCTGGTGGAAGG-3’
*Rhox5*	5’-GGATGCCTGTGTGTCCAGAGT-3’	5’-TCTAAAATGTTCAGGGACTGGTGTT-3’
*Rhox6*	5’-CCACATGTTCTGAATAGG-3’	5’-GGGCTCTCCTCATCCG-3’
*Rhox7*	5’-CCGCATACGGCCAGTTT-3’	5’-TTGGCCTCAGTCACACC-3’
*Rhox8*	5’-AGGGGTCAAGGAGAGGA-3’	5’-ACCTATCCATCTCGCGA-3’
*Rhox9*	5’-CACAGGCTGGGAACTAT-3’	5’-AGGGCTCTCCTCATCTT-3’
*Rhox10*	5’-GTGGACGAATGCAAGGT-3’	5’-TGGCACACAATGAACC-3’
*Rhox11*	5’-ATCAGTATCCAGATGCCC-3’	5’-TCTTGGATTCCACCAAAG-3’
*Rhox12*	5’-CGGATCCAGTTGGGTTTC-3’	5’-CACTTGAGACTGCTGTTC-3’
*Rhox13*	5’-GCCTGTTTGAAGAGACCCA-3’	5’-GAAGGCTCTTCAAAATCGG-3’
*Stra8*	5’-TCAACAACCTAAGGAAGGCA-3’	5’-TCCAGGCTTTCTTCCTGTTC-3’
*c-kit*	5’-TGCCTGAAACAAGTCACCTC-3’	5’-TTTACCTGGGCTATGTGCTG-3’
*Rec8*	5’-CCCCAGAATGGTGGGCCTGGT-3’	5’-AGGCCTTGGGGGCATCTCCA-3’
*Spo11*	5’-ACCCGCGTGGCCTCTAGGTTT-3’	5’-AGCATTGCTCCTCTTGGTTGCAT-3’
*Sycp1*	5’-TCTGAGGGGAAGCTCACGGTT-3’	5’-ACTTGACTGCTGCTCAGCCTCG-3’
*Sycp3*	5’-GGTTCAGAAGAAGATGTTGCTG-3’	5’-TTGTTGATGTCAGCTCCAAAT-3’
*Rad51*	5’-CAACGAAGCGCGTTCGAGCC-3’	5’-GCATAGCCATGACTGTCCCGCG-3’
*Ldhc*	5’-CGGCTTCCCTGTAGGCCGTG-3’	5’-ATGGGCACACTGGAGTCCCCA-3’
*Mvh*	5’-GTGATTCAGGCAATGGTGAC-3’	5’-CACTGCTTTCACCTCCTTCA-3’
*Crem*	5’-CACTGCCACAGGTGACATGCC-3’	5’-GGGCAGCTTCCCTGTTTTTCATCA-3’
*Acrv1*	5’-TGGATCTGCCCAAGGAGCACCA-3’	5’-CTCGGCATCTGAAGGGTTTGCGA-3’
*Acrosin*	5’-ACGTGTGATGGTCCCTGTGGGT-3’	5’-GCGTGGTACCTGCGGCTGTT-3’
*Stambp1*	5’-CCAGCTCACCGTTCTCGCCC-3’	5’-CAGGCCTTCAATGGTGGGAACATTT-3’
*Prm1*	5’-GGAGGCGAAGATGCTGCCGTC-3’	5’-TGGCGAGATGCTCTTGAAGTCTGG-3’
*Prm2*	5’-GCATCGCAGAGGCTGCAGAAGAT-3’	5’-AGAATGGACAGGCCTGGGGAGG-3’
*Tnp1*	5’-ATGTCGACCAGCCGCAAGCT-3’	5’-TGCGACTTGCATCATCGCCCC-3’
*Tnp2*	5’-AGAGGCGTAGCTCAGGGCGAA-3’	5’-CCCAACAGTCCCCTAGTGATGGC-3’
*Dazl*	5’-GACGTGGATGTGCAGAAGAT-3’	5’-GTGGTGGAGGAGGAGGATTA-3’
*Wt1*	5’-AAGGGCAGAGCAACCACGGC-3’	5’-ACCGGACAAGAGTTGGGGCCA-3’
*Cyp450*	5’-GGCCCCATTTACAGGGAGAAGCT-3’	5’-CGGGTTGGGACCCTCGCATG-3’
*Lhgcr*	5’-CCAGAGTTGTCAGGGTCGCGC-3’	5’-TGAGAGATAGTCGGGCGAGGCCAG-3’

Cytoplasmic and nuclear protein extracts from adult testis were prepared as described elsewhere [27]. Western blotting analysis was performed as described previously [28]. Various amounts of protein lysates were electrophoresed in a 12% SDS-polyacrylamide, transferred to Immobilon-P PVDF membrane (Millipore Corporation, Billerica, MA), and probed with antibodies against RHOX3 custom generated by ProteinTech Group Inc., Chicago, IL in rabbits against the N-terminal portion of RHOX3 (110 amino acids), β-actin (Sigma Chemical Co., St. Louis, MO), and Histone H3 (Abcam, Cambridge, MA). The membrane was then incubated with the secondary antibody (ECL anti-rabbit or anti-mouse from GE Healthcare UK limited) and later developed using the Super Signal West Pico Chemiluminescent substrate (Thermo Scientific, Rockford, IL). For quantification, blots were incubated with IRDye800 conjugated anti-rabbit IgG or IRDye680 conjugated anti-mouse IgG, and the signal detected using a Odyssey scanner and quantified following the manufacturer’s instruction (LI-COR biotechnology, Lincoln, ME).

### Microarray analysis

Microarray analysis was performed at the University of California, San Diego (UCSD) GeneChip microarray core facility using total cellular RNA from P15 testis isolated from *Rhox3*-shRNA mice (*Rhox3*-shRNA;*Stra8*-iCre double-transgenic mice; n = 3) and control mice (*Rhox*3-shRNA single-transgenic mice; n = 3), using an Affymetrix GeneChip mouse exon 1.0 ST array. Raw data was normalised using robust multi-array averaging with correction for GC content of the probes (GCRMA) [29]. Gene expression levels were calculated using the mean of the signal for all probe sets per gene. Changes in gene expression between control- and *Rhox3*-shRNA testes were statistically tested using one-way ANOVA. All microarray data analysis was carried out using Partek genomics suite v6.3 (Partek, St. Louis, MI, USA). Microarray data is available from Gene Expression Omnibus under accession number GSE64324.

### Phenotypic analysis and TUNEL assay

For histology analysis, testes and epididymis were fixed in either 10% neutral buffered formalin or Bouin’s fixative and stained with hematoxylin-eosin or periodic acid Schiff-hematoxylin, as previously described [5]. The size of the seminiferous tubules was determined using ImageJ software by measuring tubule size diameter in testis sections (n = 3 mice, 100 tubules total). Caudal epididymal sperm count was determined using a hemocytometer, as previously described [4]. TUNEL assays were performed on testis cross-sections with the ApopTag plus fluorescein *in situ* apoptosis detection kit (Chemicon, Temecula, CA), following the manufacturer’s instructions.

## Results

### *Rhox3* mRNA and RHOX3 protein is expressed in male germ cells

To assess the spermatogenic cell types in which the *Rhox3* gene paralogs have the potential to function, we examined their developmental expression pattern during the first wave of spermatogenesis. Using qRT-PCR analysis with primers designed to amplify all eight *Rhox3* paralogs, we found that *Rhox3* mRNA is expressed at only trace levels during early stages of the first wave of spermatogenesis—postnatal day 5 (P5) to P18—when postnatal germ cells first proliferate and undergo meiosis. *Rhox3* mRNA level is upregulated at P20 (S1A Fig.), a critical time point in which post-meiotic haploid germ cells—round spermatids—are first formed. *Rhox3* mRNA level is further elevated at later time points, corresponding to the time at which round spermatids differentiate into elongating spermatids.

**Fig 1:**
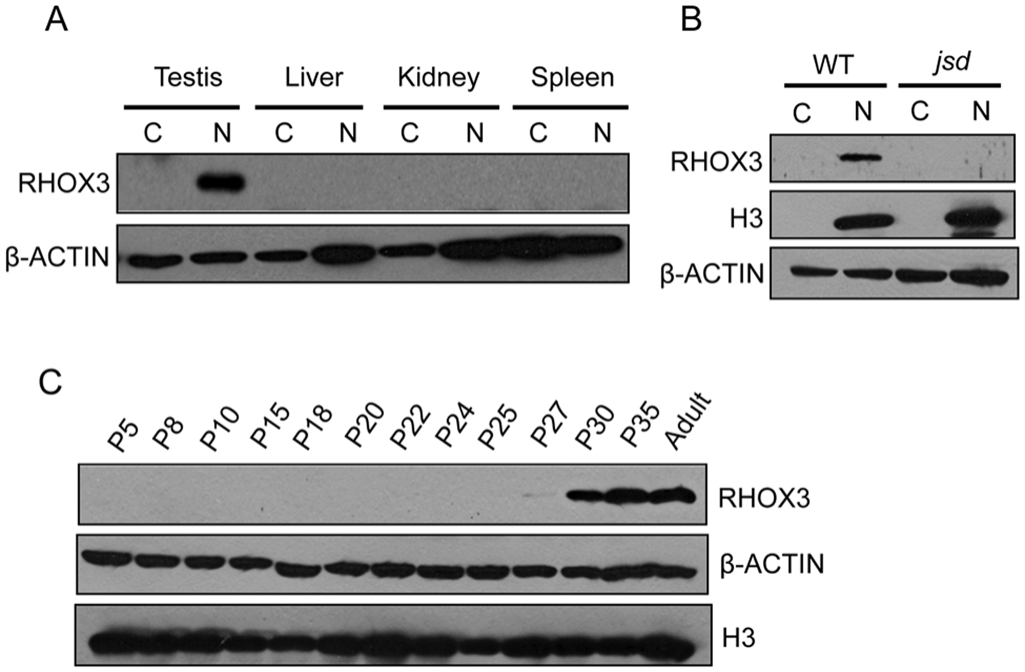
RHOX3 expression pattern in mice. Western blot analysis of adult tissue lysates (A), wild-type (WT) and *jsd* mutant mice testes lysates (B), and testes nuclear lysates from postnatal wild-type mice of the ages indicated (C). N, nuclear protein extract; C, cytoplasmic protein extract. β-ACTIN is the loading control. Histone-H3 is a chromatin marker to assess purity of the nuclear fraction.

To study the expression pattern and localization of the RHOX3 protein, we generated an antiserum against mouse RHOX3 in rabbits. For an immunogen, we chose the N-terminal half of RHOX3, which is unique to RHOX3 as it lacks the homeobox domain that is common to all homeodomain proteins and thus should elicit antibodies specific for RHOX3. Of note, this 110 amino-acid region is nearly identical in all RHOX3 paralogs, with only 0 to 4 substitutions. Western blot analysis revealed that RHOX3 antiserum detected a ~18 kDa band (the predicted size of RHOX3) in testes nuclear lysates (S1B Fig.). In addition to its size, several further lines of experimental evidence supported the notion that the ~18 kDa band is RHOX3: (i) the band was specifically detected in testes, but absent in other tissues (Fig. 1A), consistent with our previous qRT-PCR analysis showing that *Rhox3* mRNA is specifically expressed in the testis [4]; (ii) pre-immune serum derived from the rabbit before immunization did not detect the band in the nuclear fraction (S1B Fig.); (iii) the band was not detected when RHOX3 antiserum was pre-incubated with excess amounts of recombinant RHOX3 (S1B Fig.).

To assess whether RHOX3 protein is expressed in germ cells or somatic cells, we tested *jsd* mice testes, which lack virtually all germ cells except for undifferentiated spermatogonia [30]. RHOX3 was undetectable in *jsd* mutant testes, implying that RHOX3 is expressed predominantly in germ cells (Fig. 1B). To determine the developmental expression pattern of RHOX3 protein, we performed western blot analysis of testes lysates from different postnatal time points. RHOX3 protein first became detectable at P27 and increased in level thereafter, consistent with its initial expression in late round spermatids (Fig. 1C). Of note, the expression of RHOX3 protein was first detectable later during postnatal development (at P27) than the upregulation of *Rhox3* mRNA (at P20) (S1A Fig.), suggesting the possibility of translation regulation (see Discussion).

### Generation of *Rhox3*-shRNA mice

**Fig 2:**
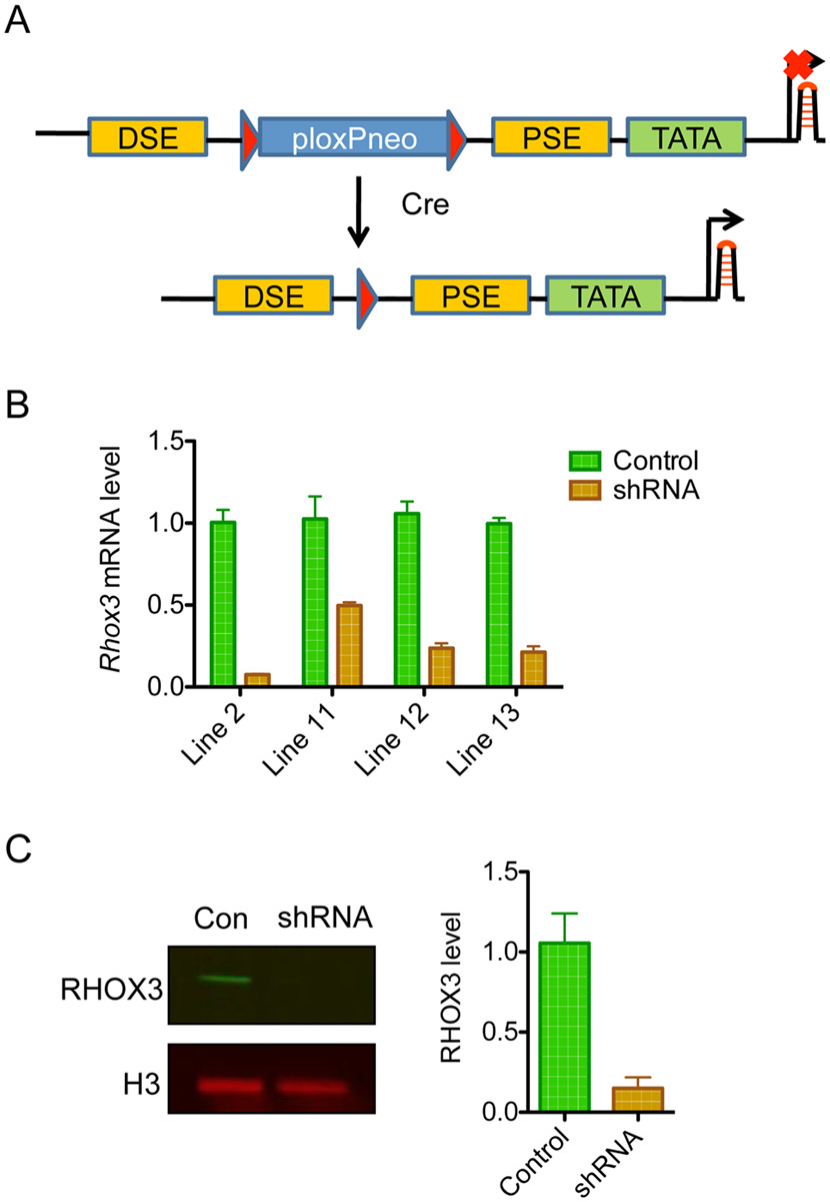
Generation and validation of *Rhox3*-shRNA mice. (A) Schematic diagram of the DNA construct used for transgenic mice generation. DSE, distal sequence element; PSE, proximal sequence element; TATA, TATA box. CRE juxtaposes the DSE and PSE to allow for transcription. (B) qRT-PCR analysis of testes from the indicated *Rhox3*-shRNA transgenic mice lines compared to littermate control mice (n = 4 for each mice line). *Rhox3* mRNA levels were normalized against the mRNA encoding the ribosomal protein RPL19. Values denote the mean fold change ±S.E. (C) Western blot analysis of nuclear testes lysates from *Rhox3*-shRNA line-2 mice (shRNA) and control mice (Con) (n = 3 for each). RHOX3 protein level was quantified using the Odyssey system and normalized to the level of Histone-H3 protein. Values denote the mean fold change ±S.E. *Rhox3*-shRNA mice, *Rhox3*-shRNA;*Stra8*-iCre double-transgenic mice; Control mice, *Rhox3*-shRNA single-transgenic mice.

To determine the functional role of RHOX3 in male germ cells *in vivo*, we elected to use a conditional RNAi approach developed by Shukla *et al*. [31]. In this approach, a short hairpin RNA (shRNA) targeting the mRNA of interest is expressed from a polymerase III promoter—the U6 promoter—which is designed to be activated by Cre recombinase (CRE). In the absence of CRE, the U6 promoter is inactive as a result of the presence of a loxP-Neo cassette between the proximal sequence element and the distal sequence element. These two elements must be adjacent for U6 promoter-dependent transcription and thus transcription is silent in the absence of CRE. Introduction of CRE removes the cassette, which juxtaposes these elements and thereby activates U6 promoter-dependent transcription (Fig. 2A).

To use this system to conditionally knockdown the expression of all eight *Rhox3* genes *in vivo*, we first identified siRNAs exhibiting 100% complementary with all eight *Rhox3* gene paralogs and then screened for those that efficiently depleted *Rhox3* expression *in vitro*. This screen led to the identification of a siRNA sequence that reduced RHOX3 protein expression by ~90% in HeLa cells upon cotransfection with an expression vector encoding FLAG-tagged mouse RHOX3 (data not shown). We inserted this siRNA sequence (in a shRNA context) into the U6-ploxP neo construct (kindly provided by from Dr. Chuxia Deng, NIH) to create a *Rhox3*-shRNA transgene construct (Fig. 2A). This transgene construct conferred strong silencing of RHOX3 protein expression in HeLa cells upon cotransfection with expression vectors encoding FLAG-tagged mouse RHOX3 and CRE (data not shown).

The *Rhox3*-shRNA transgene was injected into the pronucleus of mouse embryos to produce transgenic founder mice. Four transgenic founders expressing the *Rhox3*-shRNA transgene were produced: lines 2, 11, 12, and 13. To examine the role of *Rhox3* in male germ cells, we used *Stra8*-iCre mice, which express CRE specifically in germ cells in the testis at the initiation of spermatogenesis (between P3 and P7) [32]. *Stra8*-iCre mice were crossed with mice from the four transgenic lines to generate double transgenic mice that carry both *Stra8*-iCre and the *Rhox3*-shRNA transgene. These *Rhox3*-shRNA;*Stra8*-iCre mice (hereafter called *Rhox3*-shRNA mice) were sacrificed at 6 weeks of age and analyzed for endogenous *Rhox3* expression in the testis. The single transgenic mice that carry either the *Stra8*-iCre or *Rhox3*-shRNA transgene from same litter served as control mice. Testes from the 4 different *Rhox3*-shRNA mice lines displayed a ~50% to 85% reduction in *Rhox3* mRNA level relative to control mice testes (Fig. 2B). We examined RHOX3 protein expression in a line that had low *Rhox3* mRNA level (Line 2) and observed a dramatic decrease in RHOX3 protein level (>95%) (Fig. 2C).

### *Rhox3*-shRNA mice have dramatic spermatogenic defects

**Fig 3:**
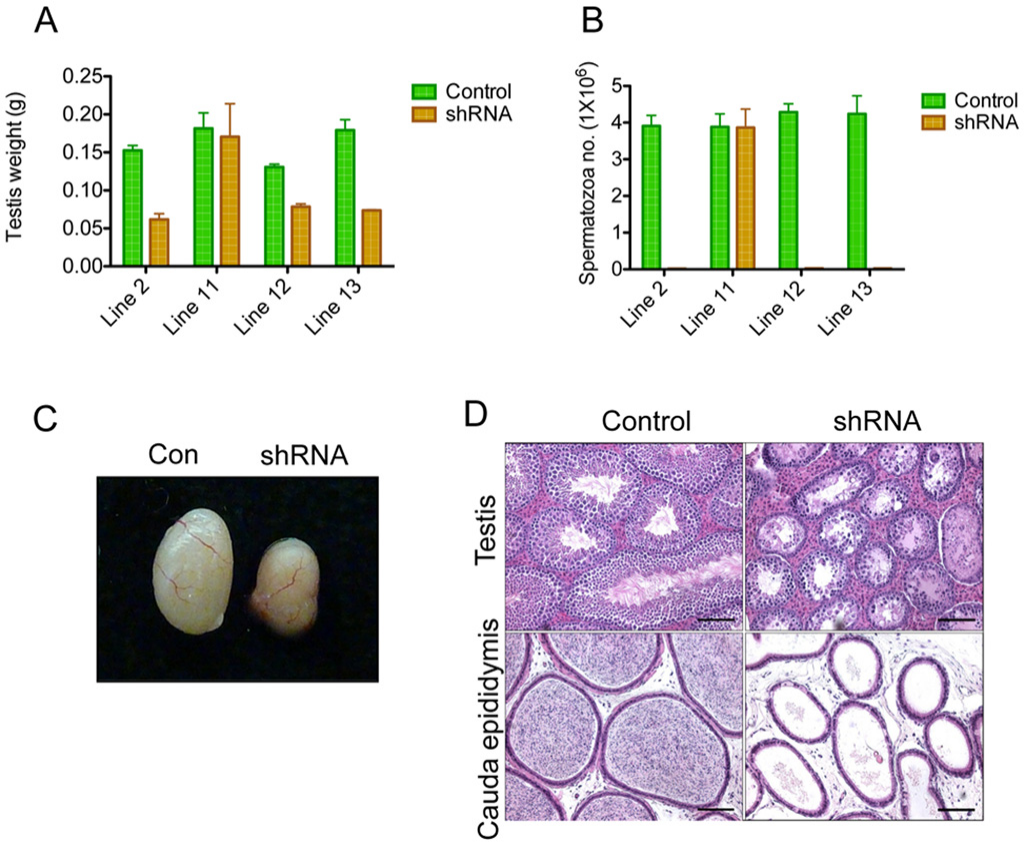
Phenotypic analysis of *Rhox3*-shRNA mice. (A) Testes weight of the control and *Rhox3*-shRNA mice at 6 weeks age derived from Lines 2, 11, 12 and 13. Values denote the mean ± S.E. (B) Sperm counts of the control and *Rhox3*-shRNA mice lines at 6 weeks of age. Values denote the mean ± S.E. (C) Representative image of testis from control and *Rhox3*-shRNA mice (Line 2) at 6 weeks age. (D) Representative images of hematoxylin and eosin (H&E) stained cross sections of testis and epididymis of control (Con) and *Rhox3*-shRNA (shRNA) mice. Scale bar: 100 μm. *Rhox3*-shRNA mice, *Rhox3*-shRNA;*Stra8*-iCre double-transgenic mice; Control mice, *Rhox3*-shRNA single-transgenic mice.

The weight of testes from adult *Rhox3*-shRNA mice lines 2, 12, and 13 decreased (by ~60%, 40%, and 60%, respectively), compared to littermate control mice that had the *Rhox3*-shRNA transgene but lacked CRE (Fig. 3A and 3C). In contrast, Line 11 did not exhibit a statistically significant reduction in testes weight compared to control mice (Fig. 3A), which is consistent with it having only a modest reduction in *Rhox3* mRNA level (Fig. 2B). None of the *Rhox3*-shRNA lines exhibited a statistically significant reduction in body weight (S2A Fig.) or seminal vesicle weight (S2B Fig.) compared to the littermate control mice. Consistent with the reduction in testis weights in Lines 2, 12, and 13, the diameter of seminiferous tubules was decreased, along with a drastic reduction in the number of post-meiotic germ cells, as described below (Fig. 3D). The average diameter of seminiferous tubules in *Rhox3*-shRNA (Line 2) was decreased by ~50% (Control vs. *Rhox3*-shRNA: 252.2±6.3 μm vs. 129.8±2.1 μm; mean±SE, n = 3). The majority of the tubules had vacuoles (Fig. 3D), perhaps forming as a result of the cytoplasmic extensions of the Sertoli cells in the absence of post-meiotic germ cells, a phenotype observed in other mice mutants, including *Dicer*-, *p53*-, and *Elmo1*-mutant mice [33–36]. Histological analysis revealed that the cauda epididymides of *Rhox3*-shRNA mice had few sperm (Fig. 3D), a finding that was confirmed by quantitative analysis (>100 fold reduction in sperm count; Fig. 3B). The dramatic reduction in sperm count suggested that such mice were infertile, which we confirmed for Line 2 (S1 Table).

**Fig 4:**
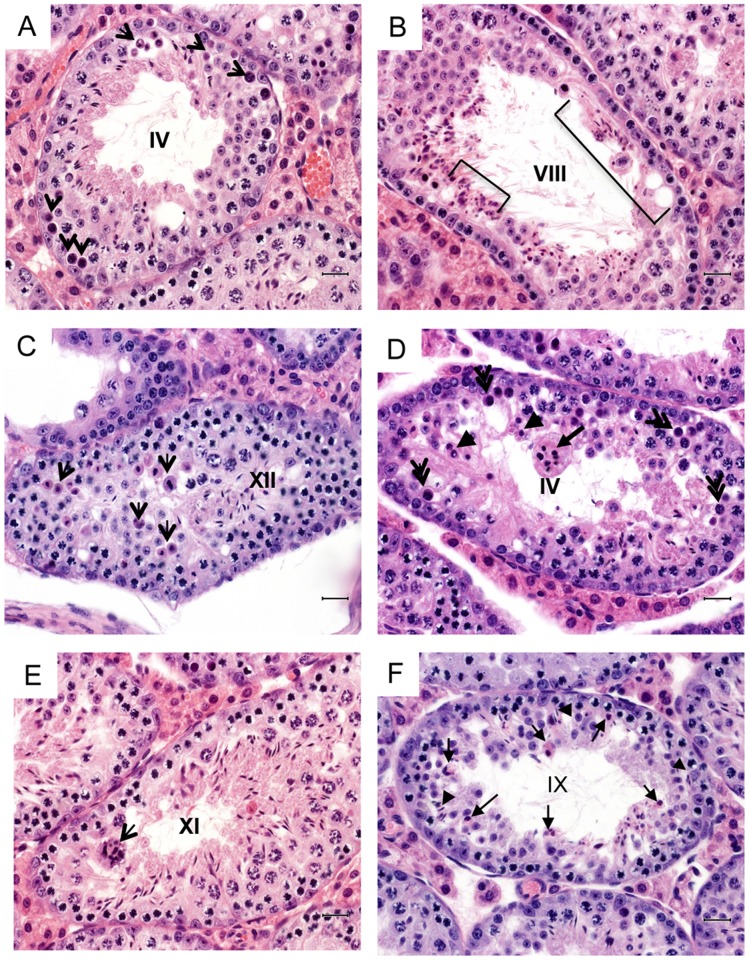
Histological analysis of *Rhox3*-shRNA mice. (A–F) Images of periodic acid Schiff (PAS)-hematoxylin stained cross-sections of adult testis from *Rhox3*-shRNA mice (Line 2). Roman numbers indicate seminiferous tubule stages. (A) Arrows, apoptotic pachytene spermatocytes. (B) Brackets, missing generations of pachytene spermatocytes and round spermatids. (C) Arrows, apoptotic metaphase spermatocytes. (D) Double arrows, apoptotic pachytene spermatocytes; arrow, a clump of apoptotic cells; arrow-heads, apoptotic round spermatids. (E) Arrow, a clump of spermatids, which are so-called “giant cells” that will probably undergo apoptosis. (F) Arrows, abnormal elongating spermatids; arrow heads, elongating spermatids abnormally deep in the epithelium. Scale bar: 25 μm. *Rhox3*-shRNA mice, *Rhox3*-shRNA;*Stra8*-iCre double-transgenic mice; Control mice, *Rhox3*-shRNA single-transgenic mice.

To examine the nature of the spermatogenesis defect, detailed histological analysis was carried on Line 2 *Rhox3*-shRNA testes. Several lines of evidence suggested that there were numerous defects in germ cell development, as early as meiotic prophase: (i) increased numbers of apoptotic pachytene spermatocytes in seminiferous epithelial cycle stage IV, leading to missing generations of spermatocytes and/or spermatids in particular tubule areas in stages VI-VIII (Fig. 4A–B); (ii) fewer pachytene spermatocytes (decreased by 63% in stage V to early VII; S2 Table); (iii) more frequent apoptotic spermatocytes undergoing meiotic metaphase in stage XII (Fig. 4C); (iv) fewer round spermatid-containing tubules (decreased by 58% in stages I to VIII; S2 Table); (v) a reduction (by 63%) in the average number of round spermatids per spermatid-containing seminiferous tubule (S2 Table). The reduced number of spermatids in the *Rhox3*-shRNA adult mice is unlikely to be exclusively the result of loss of earlier stage cells, as we observed several morphological defects in spermatids that were consistent with their degeneration and/or apoptosis. For example, *Rhox3*-shRNA mice had many multinucleated “giant cells” composed of clones of round or elongated spermatids that were clumped together and thus were likely destined for apoptosis; this phenotype was rarely observed in control mice (Fig. 4D–E). We also observed small groups of elongated spermatids with abnormal morphology, consistent with their undergoing apoptosis (Fig. 4E). Finally, we observed elongated spermatids buried deep in the seminiferous tubule epithelium that were not in a position to be released to the lumen, suggesting that they are instead destined for phagocytosis by Sertoli cells (Fig. 4F).

To further examine the nature of the germ-cell defects, we carried out qRT-PCR analysis on a battery of testicular germ cell- and somatic cell-expressed genes in adult testes from *Rhox3*-shRNA and control mice (S3 Fig.). All post-meiotic germ cell marker genes analyzed were decreased dramatically (by ~3- to 9-fold) in *Rhox3*-shRNA adult testes relative to control adult testes. Some spermatocyte marker genes (*Spo11* and *Rec8*) were also decreased significantly, but by less (~2- to 3-fold) than post-meiotic genes. Other spermatocyte marker genes (*Sycp1* and *Sycp3*) did not exhibit a significant change in level. This confirmed that there was a defect in the progression of spermatocytes, probably at a mid or late phase. In contrast, spermatogonia markers (*Stra8* and *c-Kit*) were increased in level by ~2- to 3-fold in *Rhox3*-shRNA testes, which is consistent with an enrichment of spermatogonia as a result of depletion of spermatocytes and spermatids. Similar increases were observed for Sertoli cell markers (*Wt1* and *Rhox5*) and Leydig cell markers (*Cyp450* and *Lhgcr)*, suggesting that these cell populations were enriched too. Similar findings were observed in mid-postnatal (P20 and P25) testis samples (S4B–C Figs.). In contrast, no significant alteration in germ cell marker expression was observed in early postnatal (P15) testis, a stage in which the most advanced germ cells have only reached mid-meiosis (S4A Fig.). Together, these data suggest that the *Rhox3*-shRNA testes have one or more defects in spermatocytes undergoing progression through meiotic prophase.

### *Rhox3* shRNA causes spermatogenic defects independently of RHOX3 depletion

We were surprised that *Rhox3*-shRNA mice had defects in spermatocytes given that the *Rhox3* gene paralogs are likely to be transcriptionally repressed by MSCI in spermatocytes (S1 Fig.) and RHOX3 protein is not detectable until P27 (Fig. 1C), well after the first wave of meiosis (~P10 to P20). Indeed, RHOX3 protein is not robustly expressed until P30 (Fig. 1C), when male germ cell maturation reaches its final stage—the conversion of elongating spermatids into elongated spermatids. This raised the possibility that the dramatic spermatogenic defects in *Rhox3*-shRNA mice might not result from depletion of RHOX3, but rather from an off-target effect. We first considered the possibility that the *Rhox3* shRNA cross-regulates one or more other members of the *Rhox* gene cluster that are expressed in spermatocytes, as this would explain why we observed reduced numbers of spermatocytes in the *Rhox3*-shRNA mice. However, qRT-PCR analysis showed that the expression of other *Rhox* cluster genes was not significantly reduced in *Rhox3*-shRNA mice testes (S4D Fig.). Thus, we deem it unlikely that the defects result from cross-regulation of other *Rhox* cluster genes.

**Fig 5:**
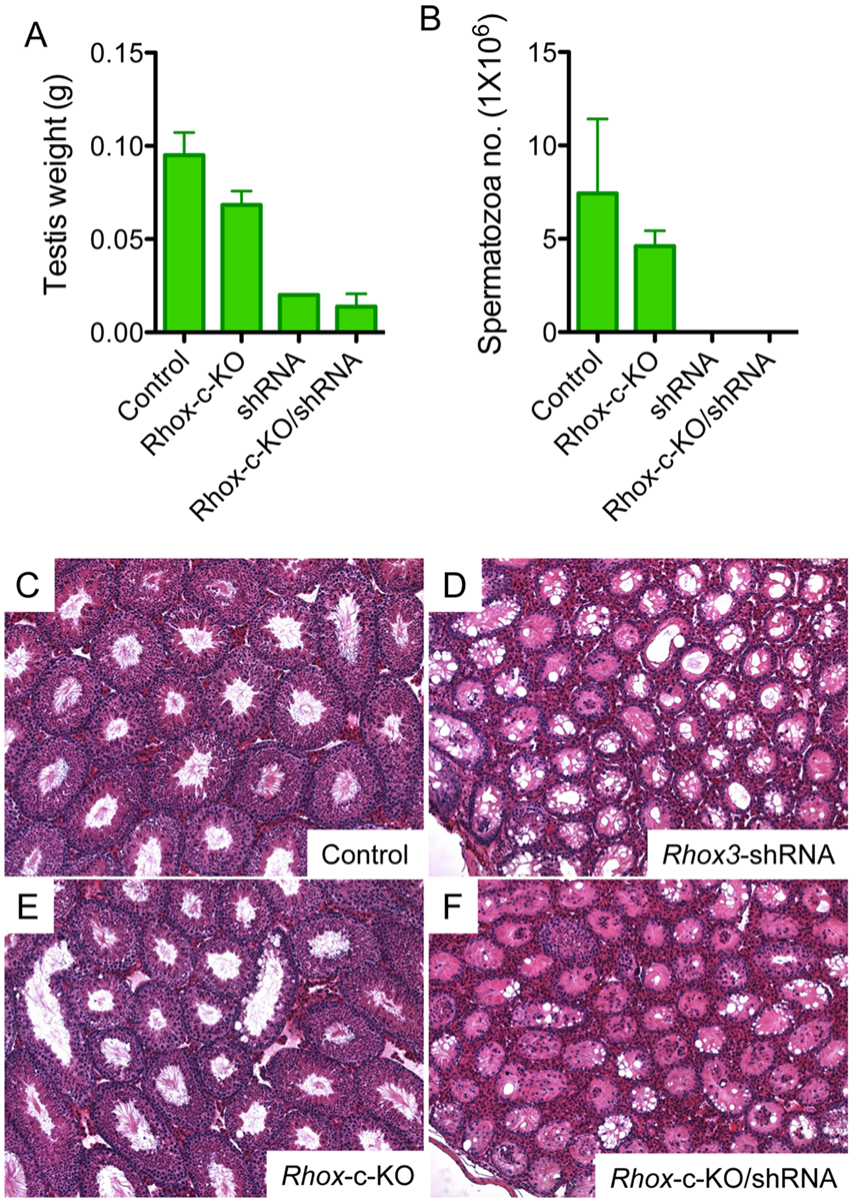
*Rhox3* independent phenotype of *Rhox3*-shRNA mice. (A, B) Testis weight (A), and sperm number (B) of control, *Rhox*-c-KO, *Rhox3*-shRNA (shRNA) and *Rhox*-c-KO/*Rhox3*-shRNA (*Rhox*-c-KO/shRNA) mice at 7–12 weeks of age. Values denote the mean ± S.E. (C-F) Representative images of hematoxylin and eosin (H&E) stained testis cross-sections from control (C), *Rhox3*-shRNA (D), *Rhox*-c-KO (E) and *Rhox*-c-KO/*Rhox3*-shRNA mice (F). *Rhox3*-shRNA mice, *Rhox3*-shRNA;*Stra8*-iCre double-transgenic mice; Control mice, *Stra8*-iCre single-transgenic mice; *Rhox*-c-KO mice, *Rhox* cluster-KO mice; *Rhox*-c-KO/*Rhox3*-shRNA mice, *Rhox*-c-KO;*Rhox3*-shRNA;*Stra8*-iCre mice.

To directly assess whether the defect is an off-target effect, we made use of mice lacking the entire *Rhox* cluster, including all eight *Rhox3* paralogs. The generation of these mice is described in the Materials and Methods; their full phenotypic analysis will be provided elsewhere (Song *et al*., manuscript in preparation). In brief, we introduced loxP sites at both ends of the *Rhox* cluster in ES cells (by homologous recombination), generated mice harboring these loxP sites, and mated them with *EIIa*-Cre mice (which express Cre from a ubiquitous promoter) to generate *Rhox*-cluster-KO (*Rhox-c*-KO) mice progeny. These *Rhox-c*-KO mice did not exhibit the defect in meiotic prophase we detected in *Rhox3*-shRNA mice, but instead had only a modest (~28%) reduction in testes size (Fig. 5A), a minor reduction in the number of all stages of germ cells with no obvious histological abnormality in seminiferous tubules (Fig. 5E), and a modest reduction in tubule diameter compared to littermate control mice at 6 weeks of age (Fig. 5E).

The much more modest spermatogenic defects in 6-week old mice lacking the entire *Rhox* cluster in germ cells than in mice expressing the *Rhox3* shRNA was consistent with the *Rhox3* shRNA exerting an off-target effect. However, other explanations are possible. For example, *Rhox3*-shRNA mice may have severe defects because depletion of RHOX3 upsets a delicate balance between *Rhox* genes that must be in place for normal spermatogenesis. To test whether or not depletion of RHOX3 is responsible for the dramatic spermatogenic defects observed in *Rhox3*-shRNA mice, we bred these mice with *Rhox-c*-KO mice to generate *Rhox-c*-KO/*Rhox3*-shRNA mice (described in detail in the Materials and Methods). If the *Rhox3*-shRNA mice phenotype is the result of depletion of *Rhox3*, *Rhox-c*-KO/*Rhox3*-shRNA mice would be predicted to show the same defect as *Rhox*-c-KO mice because *Rhox3* is not there to be depleted by shRNA. Alternatively, if the defect from the shRNA is independent of the presence of *Rhox3*, *Rhox*-c-KO/*Rhox3*-shRNA mice would be predicted to show the same severe defect as *Rhox3*-shRNA mice. We found that the *Rhox-c*-KO/*Rhox3*-shRNA mice showed virtually the same phenotype as that of *Rhox3*-shRNA mice: 80% reduction in testis weight (Fig. 5A), dramatic reduction in sperm number (Fig. 5B), dramatic reduction of post-meiotic germ cells (Fig. 5F), and severe histological abnormalities in seminiferous tubules (Fig. 5F). Since these *Rhox3*-shRNA expressing mice lack the *Rhox3* gene paralogs, this means that their spermatogenic defects could not be caused by depletion of RHOX3.

### *Rhox3*-shRNA mice have reduced levels of endogenous siRNAs

What is responsible for the severe spermatogenic defects in the mice expressing the *Rhox3* shRNA? One possibility is that it results from an off-target effect of the *Rhox3* shRNA on one or more genes that function in spermatogenesis. To assess this, we performed microarray analysis of *Rhox3*-shRNA vs. control testes at a postnatal time point (P15) that corresponds to mid-meiosis. The time point is ~10 days after *Rhox3* shRNA would be predicted to be first expressed as a result of CRE-mediated transcriptional activation [32]. We also chose the P15 time point, as there were no detectable germ cell composition alterations in *Rhox3*-shRNA mice at this developmental time point (S4A Fig.). In contrast, germ cell subset alterations were noticeable by P20 (S4B Fig.), which would have complicated interpretation of gene expression data. Microarray analysis revealed only one gene that exhibited a >2-fold change in expression level (S3 Table). In total, 34 genes exhibited significantly increased expression and 8 genes exhibited significantly decreased expression in *Rhox3*-shRNA testes relative to littermate control testes (>1.5 fold up or down; P<0.05; S3 Table). While it is possible that one or more of the 8 down-regulated genes are decreased in level as a result of an off-target effect of the *Rhox3* shRNA, none are dysregulated by more than 50% and none are predicted to be targeted by the *Rhox3* shRNA, as defined using Blast software. We also tested another potential mechanism—that *Rhox3* shRNA acts like a miRNA on 3’ UTRs via partial complementarity [37]. Using RNAhybrid software (http://bibiserv.techfak.uni-bielefeld.de/rnahybrid/), we found that only one of the 8 down-regulated genes exhibit complementarity with the putative *Rhox3* shRNA seed site—position 2–8. This gene, *Cldn2*, encodes a member of the claudin family of proteins, which are considered to be the most important components of tight junctions. While some claudins have been shown to function in spermatogenesis, *Cldn2* does not appear to have such a role, as *Cldn2*-KO mice have only been reported to have kidney dysfunction; no fertility defects were observed [38].

**Fig 6:**
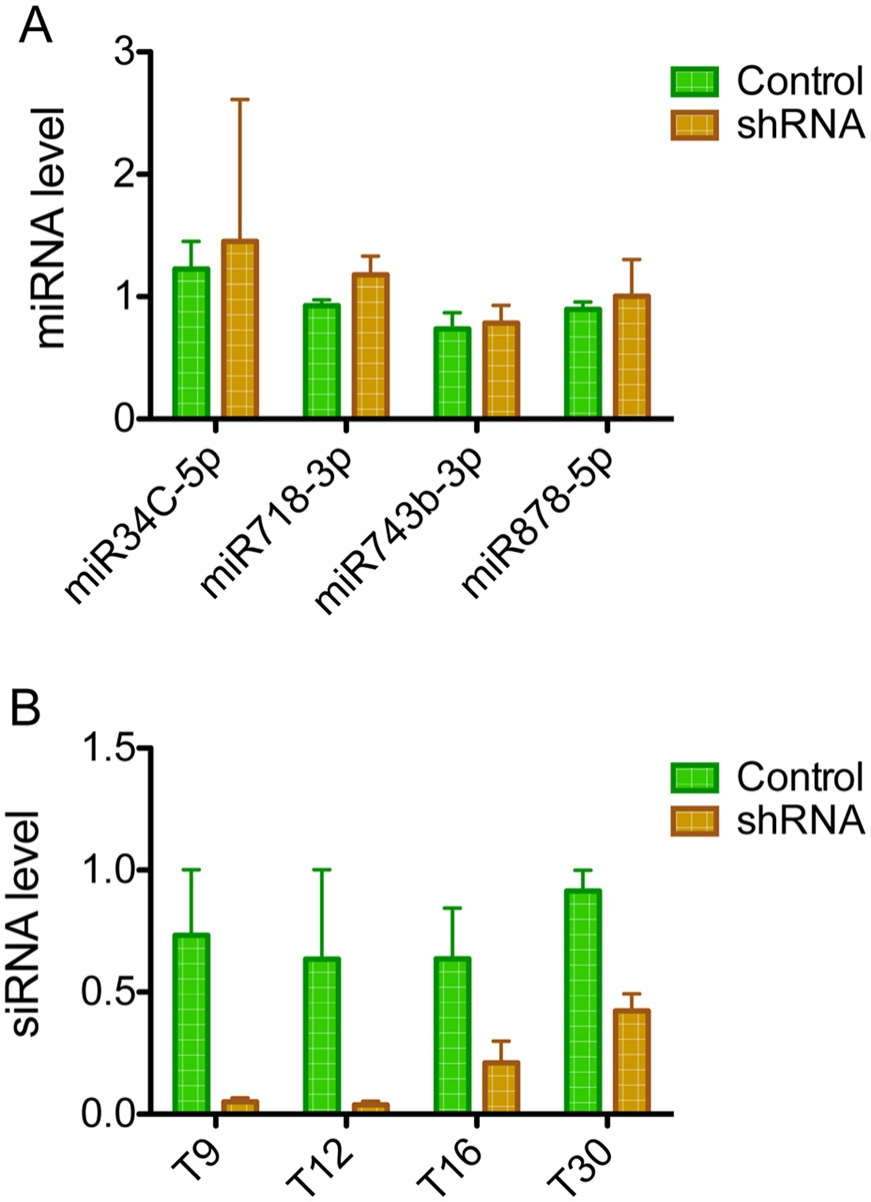
*Rhox3*-shRNA mice have reduced expression of endo-siRNAs but not miRNAs. (A) Relative miRNA levels in testes of 15 days-old control and *Rhox3*-shRNA mice. (B) Relative endo-siRNA levels in testes of 15 days old control and *Rhox3*-shRNA mice. Values denote the mean fold change ±S.E. *Rhox3*-shRNA mice, *Rhox3*-shRNA;*Stra8*-iCre double-transgenic mice; Control mice, *Rhox3*-shRNA single-transgenic mice.

Finally, we considered another potential mechanism—that processing of the *Rhox3* shRNA ties up a sufficient proportion of the small RNA processing machinery that it reduces the generation of small RNAs. We considered this to be an attractive possibility, as the histological defects we observed in *Rhox3*-shRNA testes were very similar to that of Dicer-conditional KO mice generated using the same Cre-driver mice as we used in our study—*Stra8*-iCre mice [33,39]. To test this possibility, we first examined the levels of miRNAs known to be expressed in spermatocytes (miR-34C, miR-743, and miR-878) and spermatogonia (miR-718) [33, 40] by Taqman analysis. None of these four miRNAs exhibited significantly altered expression in testes from *Rhox3*-shRNA mice (Fig. 6A). This indicated the *Rhox3* shRNA did not globally disrupt miRNA biogenesis.

Male germ cells are among the few cell types in mammals that express not only miRNAs, but also another class of small RNAs—endo-siRNAs. While endo-siRNAs are best known for being expressed in lower organisms, they are expressed in the early mammalian embryo and ovary of mice [21–23] and were recently also found to be expressed in testicular germ cells [20]. This raised the possibility that the *Rhox3* shRNA interferes with endo-siRNA expression in the testis. Therefore, we analyzed the level of four siRNAs (T9, T12, T16 and T30) out of 78 known testicular endo-siRNAs [20]. We chose these four because they served as representatives of the different expression classes of siRNAs assigned based on their expression pattern in postnatal testes: T9 and T12 are in spermatogonia and spermatocytes, T16 is in spermatocytes and spermatids, and T30 is in all stages of germ cells [20]. We found that all four of these testicular endo-siRNAs were significantly reduced in level (some dramatically so) in *Rhox3*-shRNA testes compared to littermate control (Fig. 6B). This strongly suggested that *Rhox3*-shRNA expression in germ cells interferes with either endo-siRNA generation and/or stabilization. Given that our *Rhox3*-shRNA mice had strong spermatogenic defects, including reduced progression of spermatocytes through meiosis, this also raised the possibility that the reduced expression of endo-siRNAs in these mice is responsible for some or all of their spermatogenic defects. The function of endo-siRNAs in spermatogenesis is not currently known.

## Discussion

shRNAs are widely used to knockdown the expression of specific genes and thereby elucidate their functions. Here, we demonstrate that an shRNA can cause dramatic defects independently of its intended targets. In particular, we showed that an shRNA targeting a set of highly related homeobox genes causes severe spermatogenesis defects independently of the shRNA’s ability to repress the expression of these homeobox genes. To demonstrate this, we generated mice expressing the *Rhox3* shRNA but lacking the targets—the *Rhox3* paralogs. These *Rhox*-c-KO/*Rhox3*-shRNA mice exhibited the same severe spermatogenic defects as *Rhox3+* mice expressing the *Rhox3* shRNA (Fig. 5).

We tested several possible explanations for the off-target effects of the *Rhox3* shRNA. Our evidence did not support most of these mechanisms. First, we found that genes in the *Rhox* cluster besides *Rhox3* were not significantly mis-regulated in *Rhox3*-shRNA mice (S4D Fig.), ruling out the possibility of cross-regulation of other *Rhox* family members. Second, microarray analysis revealed that few genes were significantly dysregulated in postnatal *Rhox3*-shRNA mice testes compared to control mice testes, and most of these few were dysregulated by a very modest degree (S3 Table). While it is possible that modestly dysregulated genes are responsible for the dramatic spermatogenic defects in *Rhox3*-shRNA mice, we regard this as unlikely, as none of the downregulated genes had sequences predicted to be targeted by the *Rhox3* shRNA, even under relaxed stringency. We also did an *in silico* search for “miRNA-like” targets of the *Rhox3* siRNA and found only one potential target, *Cldn2*, a gene encoding a gap junction factor whose loss was previously shown to result in kidney defects, but no fertility defects were reported [38]. Third, the *Rhox3* shRNA transgene is unlikely to have fortuitously disrupted a gene critical for spermatogenesis by insertional mutagenesis, as we observed the same defects in several independent transgenic lines (lines 2, 12 and 13). The line with no obvious defects (line 11) exhibited only modest *Rhox3* depletion, suggesting a dose-response relationship rather than an insertional mutagenesis mechanism (Fig. 3).

The only defect we identified in the *Rhox3*-shRNA mice that is a candidate to be responsible for the spermatogenic defects is the processing of endogenous small RNAs. We originally considered this possibility because it has been previously reported that high levels of shRNAs inhibit miRNA biogenesis [41,42]. Evidence suggests that shRNAs have this effect because their processing diverts components of the miRNA processing machinery (i.e., EXPORTIN5 and/or RNA-induced silencing complexes) away from endogenous miRNA precursors and thereby impairs miRNA processing [41–43]. However, we did not observe a defect in the biogenesis of the miRNAs we tested; instead we found that *Rhox3*-shRNA mice testes had greatly reduced levels of another class of small RNAs—endo-siRNAs (Fig. 6). This off-target effect of an shRNA has not been previously reported, presumably because past studies have been conducted on cell types not known to express endo-siRNAs. Indeed, the only mammalian cell types so far demonstrated to express endo-siRNAs are germ cells and embryonic stem cells [20–23]. In oocytes, endo-siRNAs are implicated in having roles in meiotic progression, as loss of factors critical for siRNA processing, DICER and AGO2, causes oocyte meiotic progression defects [44–46]. While miRNA maturation is also driven by DICER and AGO2, it is unlikely that this meiotic progression defect is caused by loss of miRNAs, as oocytes lacking DGCR8, which is required for miRNA biogenesis but not siRNA biogenesis, exhibit normal meiotic progression [47]. Furthermore, miRNA activity is globally suppressed in oocytes, suggesting that miRNAs have little or no function in oocytes [48].

The phenotype of *Rhox3*-shRNA mice resembles that of other mutant mice exhibiting defective small RNA production; i.e. as a result of conditional loss of *Dicer* in germ cells using the same Cre mice as used by our study—*Stra8*-iCre mice [20,33,39,49]. Both these *Dicer*-conditional KO mice and *Rhox3*-shRNA mice exhibited (i) reduced testes weight (Fig. 3A), (ii) reduced numbers of pachytene spermatocytes (Fig. 4, S2 Table), (iii) few or no elongated spermatids (Fig. 4), (iv) numerous vacuoles in the seminiferous tubule epithelium (Fig. 4), (v) multinucleated “giant” cells (Fig. 4), (vi) increased germ cell apoptosis (S5 Fig.), and (vii) a dramatic drop in sperm counts (Fig. 3B). Our finding that *Rhox3*-shRNA mice have defects in both endo-siRNA expression and meiotic progression provides correlative evidence that endo-siRNAs are involved in meiosis and/or spermatocyte survival. Consistent with this possibility, endo-siRNAs promote the meiotic progression of oocytes, as described above. Hundreds of predicted targets have been identified for testes-expressed endo-siRNAs and some of them have been demonstrated to reduce the expression of specific genes via their 3’ UTRs *in vitro*[20]. Coupled with our data, this supports a model in which endo-siRNAs repress key target genes to promote meiotic progression and germ cell survival in the testis. For example, it is possible that many of the 34 genes exhibiting significantly increased expression in *Rhox3*-shRNA testes (relative to littermate control testes, S3 Table) are endo-siRNA-repressed genes that are de-repressed in our transgenic mice. In support of the notion that a large fraction of these genes are regulated by endo-siRNAs, we found that ~4 times more genes were upregulated than downregulated in *Rhox3*-shRNA testes (S3 Table).

A surprising finding in our study is that siRNAs, but not miRNAs, were dysregulated by the *Rhox3* shRNA (Fig. 6), as both of these small RNAs depend on some of the same factors (i.e., DICER and EXPORTIN-5) for their processing [50]. In *Drosophila melanogaster*, there are two different DICER proteins; one devoted to processing siRNAs and the other that processes miRNAs. In contrast, there is only one known DICER protein in mammalian cells and it processes both siRNAs and miRNAs. In the future, it will be interesting to determine whether the proteins that associate with mammalian DICER to process siRNAs and miRNAs differ and whether such differences are responsible for the selective effects we observed. Of note, it has been shown that double-stranded (ds) RNA-binding proteins can change the substrate recognition and processing behavior of DICER *in vitro* [51]. For example, the interaction of protein kinase R-activating protein with DICER promotes a preference for pre-miRNA processing over shRNA processing [51]. shRNA processing is very similar to that of endo-siRNAs, as both involve processing of double-stranded RNAs or hairpin-loop RNAs [20], raising the possibility that shRNAs and endo-siRNAs compete with each other for a class of DICER complex, while microRNA-specific DICER complexes are free to efficiently process pre-miRNAs.

Another possibility is that shRNAs promote the destabilization of endo-siRNAs rather than inhibiting endo-siRNA biogenesis. This destabilization could be specific for endo-siRNAs, not miRNAs, explaining the selectivity we observed. For example, shRNAs might interfere with siRNAs forming RNA-induced silencing complexes (RISC), leading to their destabilization. In support of this, loss of AGO2, a key RISC component, results in decreased levels of siRNAs in mouse oocytes [21]. Little is known about mammalian endo-siRNA RISCs; they may differ from miRNA RISCs, explaining the differential effects on siRNA and miRNA levels we observed [22,50,52]. Two of the endo-siRNAs expressed at reduced level in *Rhox3*-shRNA mice testes (T9 and T12) are not reduced in level in *Dicer* KO pachytene spermatocytes, suggesting that some siRNAs are processed in a DICER-independent manner [20]. This, in turn, suggests that the *Rhox3* shRNA might sequester factors required for siRNA biogenesis other than DICER. Regardless of the precise mechanism, we suggest that the ability of shRNAs to inhibit the expression of endo-siRNAs *in vivo* can be used as a tool to study the biogenesis, stability, and/or function of these small RNAs.

We note that it is possible that the 4 miRNAs we demonstrated were normally expressed in *Rhox3*-shRNA mice may not be representative of all miRNAs; i.e., there may be a pool of shRNA-resistant miRNAs in male germ cells. We regard this is unlikely, as we tested endo-siRNAs with different expression characteristics (see Results) and found that all were downregulated in *Rhox3*-shRNA mice (Fig. 6B). Nevertheless, it has been shown that there are several alternative miRNA processing pathways, including both DICER- and DROSHA-independent pathways, and thus it is possible that one or more of these pathways is shRNA sensitive [23,53–55]. Thus, it will be important in the future to analyze the complete expression profile of small non-coding RNAs in *Rhox3*-shRNA mice using genome-wide RNAseq analysis. If one or more classes of miRNAs are shown to be dysregulated in *Rhox3*-shRNA mice, this will lead to studies to understand the underlying mechanism, which will ultimately provide insight into how miRNA classes differ with regard to their biosynthesis and/or stability.

While our study did not achieve our intended goal of identifying the *in vivo* functions of the *Rhox3* gene paralogs, we did define various aspects of their expression pattern. For example, we showed that, as a group, they are expressed primarily in the testis. Their postnatal expression pattern suggests they are highly expressed in round and elongating spermatids. (S1 Fig.). They are predominantly expressed in germ cells, as we could not detect RHOX3 protein in germ cell-deficient mice (Fig. 1B). The low expression of the *Rhox3* gene paralogs during the mid-phase of the first wave of spermatogenesis (P12 to P20) is consistent with them being subject to meiotic sex chromosome inactivation (MSCI), a transcriptional repression mechanism that transcriptionally silences the X and Y chromosomes during the meiotic phase of spermatogenesis [56]. After meiosis, sex chromosome repression is often maintained, explaining why sex chromosomes appear to be largely heterochromatic after meiosis, a phenomenon called post-meiotic sex chromatin (PMSC) [57]. However, PMSC is not complete; it is estimated that ~18% of X-linked genes are expressed in post-meiotic germ cells, the majority of which are part of multi-copy gene families [58,59]. Our finding that the *Rhox3* gene paralogs exhibit induced expression at a postnatal time point when round spermatids first appear (P20; S1A Fig.), strongly suggests that they are among the multi-copy X-linked gene families that escape PMSC. Of note, because the *Rhox3* gene paralogs are almost identical in sequence, our qPCR analysis did not distinguish between them; it will be interesting in the future to determine whether individual *Rhox3* gene paralogs have subtle differences in their expression pattern. Our Western blot analysis indicated that RHOX3 protein is only detectable within the nuclear fraction of testes lysates (Fig. 1), consistent with the RHOX3 proteins acting as homeobox transcription factors. Their postnatal expression pattern raises the possibility that they regulate genes important for spermatid maturation and/or differentiation into spermatozoa. Interestingly, RHOX3 proteins were first detectable considerably later during postnatal development (at P27) than the upregulation of *Rhox3* mRNA (at P20) (S1A Fig.), raising the possibility that *Rhox3* mRNAs are translationally silenced until the late round spermatid stage. By analogy, another member of *Rhox* gene cluster, *Rhox13*, is developmentally regulated at the translational level in germ cells; albeit at the spermatogonia stage [60].

Other studies in addition to ours have identified unintended effects of shRNAs. For example, target-independent cellular toxicity and animal morbidity have been shown to result from the expression of some exogenous shRNAs [61,62]. Some studies have identified candidate mechanisms responsible for off-target effects, including transcript silencing [17] and interference with endogenous miRNA functions [41,42,63]. While these studies have provided evidence for a biological phenotype being caused by an off-target effect, in most cases this was not proven. In our study, we used a novel approach to definitively demonstrate that an shRNA elicits off-target biological defects. Moreover, our study is the first to report that shRNAs can lead to endo-siRNA dysregulation. In the future, it will be important to develop shRNA-knockdown approaches that selectively act on the intended target and not endo-siRNAs. While only germ cells and embryonic stem cells have been shown to express endo-siRNAs [20–23], future studies may reveal physiologically significant expression of endo-siRNAs in other cell types. An approach worth considering to reduce off-target effects is the recently developed “targeted single copy-shRNA expression” system [64]. By expressing the shRNA from a single copy plasmid, shRNA expression will tend to be lower than the traditional multi-copy approach, thereby reducing the risk of endo-siRNA suppression. Another alternative is to generate siRNAs from Pol II-dependent miRNA precursors, rather than shRNAs, as this has been shown to largely eliminate shRNA-mediated toxicity [62]. A promising recent approach to silence genes in germ cells is to insert complementary sequence of them into piRNA-generating loci [65]. In conclusion, the growing evidence that shRNAs can have unintended effects *in vivo* highlights the need for controls to properly interpret shRNA-mediated knockdown studies. New strategies must be developed to specifically knockdown genes with reduced side effects.

## Supporting Information

S1 Fig *Rhox3* gene expression pattern in mice testes and RHOX3 antibody validation. (A) qRT-PCR analysis of *Rhox3* mRNA level (normalized to *Rpl19* mRNA) in total testicular RNA from mice (n = 3) of the age shown (*Rhox3* mRNA level at P5 was arbitrarily set to 1). Values denote the mean fold change ±S.E. (B) Western blot analysis of adult testis lysate. The blot with lanes 1 and 2 was probed with purified RHOX3 IgG (α-RHOX3). The arrow points to the presumptive RHOX3 band (~18 kDa); the higher and lower migrating bands are proteins binding non-specifically to the RHOX3 IgG. As negative controls, blots were probed with IgG purified from the preimmune serum (Pre IgG) (Lane 3) or purified RHOX3 IgG that were preincubated with excess amounts of recombinant RHOX3 protein (α-RHOX3+recRHOX3) (Lane 4). Red boxes denote the lack of detectable RHOX3 in the negative controls. β-ACTIN is the loading control. N, nuclear protein extract; C, cytoplasmic protein extract.
(TIF)

S2 Fig Phenotypic analysis of adult *Rhox3*-shRNA mice. (A) Body weight of *Rhox3*-shRNA and control mice at 6 weeks of age. (B) Seminal vesicle weight of the *Rhox3*-shRNA and control mice lines at 6 weeks of age. Values denote the mean ± S.E. *Rhox3*-shRNA mice, *Rhox3*-shRNA;*Stra8*-iCre double-transgenic mice; Control mice, *Rhox3*-shRNA single-transgenic mice.
(TIF)

S3 Fig Marker analysis of adult *Rhox3*-shRNA mice. Adult *Rhox3*-shRNA mice testes have elevated levels of spermatogonial and somatic cell markers and reduced levels of some meiotic and all post-meiotic germ cell markers. qRT-PCR analysis of testes from *Rhox3*-shRNA and control mice at 6 weeks of age. Values were normalized to the mRNA encoding the ribosomal protein RPL19 and denote the mean ± S.E. *Rhox3*-shRNA mice, *Rhox3*-shRNA;*Stra8*-iCre double-transgenic mice; Control mice, *Rhox3*-shRNA single-transgenic mice.
(TIF)

S4 Fig Marker and *Rhox* expression analysis of postnatal *Rhox3*-shRNA mice. (A–D) qRT-PCR of testes from *Rhox3*-shRNA and control mice from the indicated postnatal ages. Values were normalized to the mRNA encoding the ribosomal protein RPL19 and denote the mean ± S.E. Asterisk (*) indicates significant changes, p<0.05. *Rhox3*-shRNA mice, *Rhox3*-shRNA;*Stra8*-iCre double-transgenic mice; Control mice, *Rhox3*-shRNA single-transgenic mice.
(TIF)

S5 Fig TUNEL assay in postnatal *Rhox3*-shRNA mice testes. (A) Representative images of TUNEL assay of control and *Rhox3*-shRNA testes (P25). (B) Quantification of the percentage of seminiferous tubules containing TUNEL-positive cells at the indicated developmental stages. Asterisk (*) indicates significant changes, p<0.05. *Rhox3*-shRNA mice, *Rhox3*-shRNA;*Stra8*-iCre double-transgenic mice; Control mice, *Rhox3*-shRNA single-transgenic mice.
(TIF)

S1 Table Fertility of *Rhox3*-shRNA Male Mice. (DOCX)

S2 Table Histological Analysis of *Rhox3*-shRNA Testes. (DOCX)

S3 Table Microarray Analysis of *Rhox3* shRNA-Regulated Genes. (DOCX)
