# Supplementary figures and images for: shRNA Off-Target Effects In Vivo: Impaired Endogenous siRNA Expression and Spermatogenic Defects
Source: PLoS One. 2015 Mar 19;10(3):e0118549. doi: 10.1371/journal.pone.0118549 (PMC4366048; doi:10.1371/journal.pone.0118549)

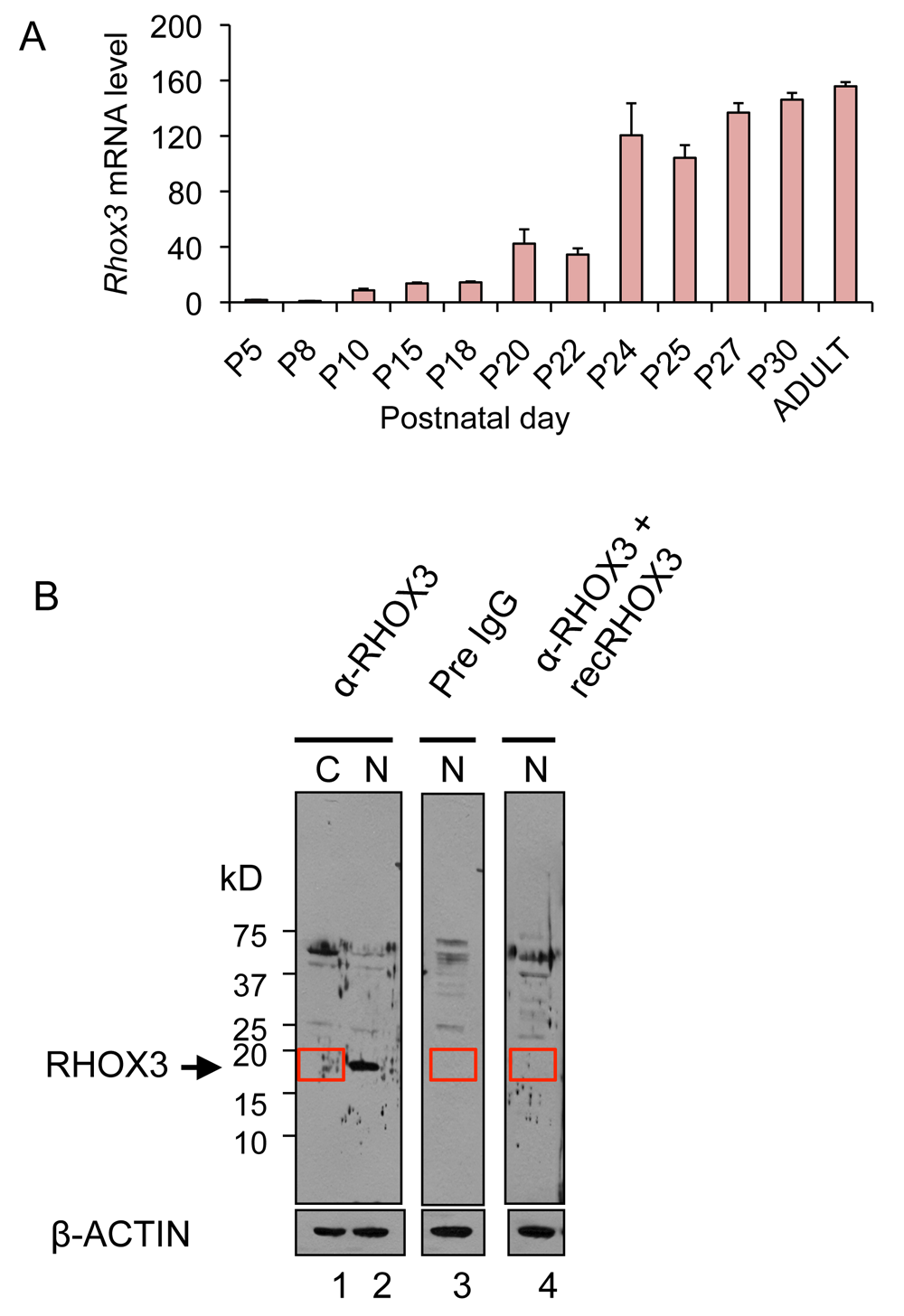

Supplement: S1 Fig — (A) qRT-PCR analysis of Rhox3 mRNA level (normalized to Rpl19 mRNA) in total testicular RNA from mice (n = 3) of the age shown (Rhox3 mRNA level at P5 was arbitrarily set to 1). Values denote the mean fold change ±S.E. (B) Western blot analysis of adult testis lysate. The blot with lanes 1 and 2 was probed with purified RHOX3 IgG (α-RHOX3). The arrow points to the presumptive RHOX3 band (~18 kDa); the higher and lower migrating bands are proteins binding non-specifically to the RHOX3 IgG. As negative controls, blots were probed with IgG purified from the preimmune serum (Pre IgG) (Lane 3) or purified RHOX3 IgG that were preincubated with excess amounts of recombinant RHOX3 protein (α-RHOX3+recRHOX3) (Lane 4). Red boxes denote the lack of detectable RHOX3 in the negative controls. β-ACTIN is the loading control. N, nuclear protein extract; C, cytoplasmic protein extract. (TIF) [file pone.0118549.s001.tif]

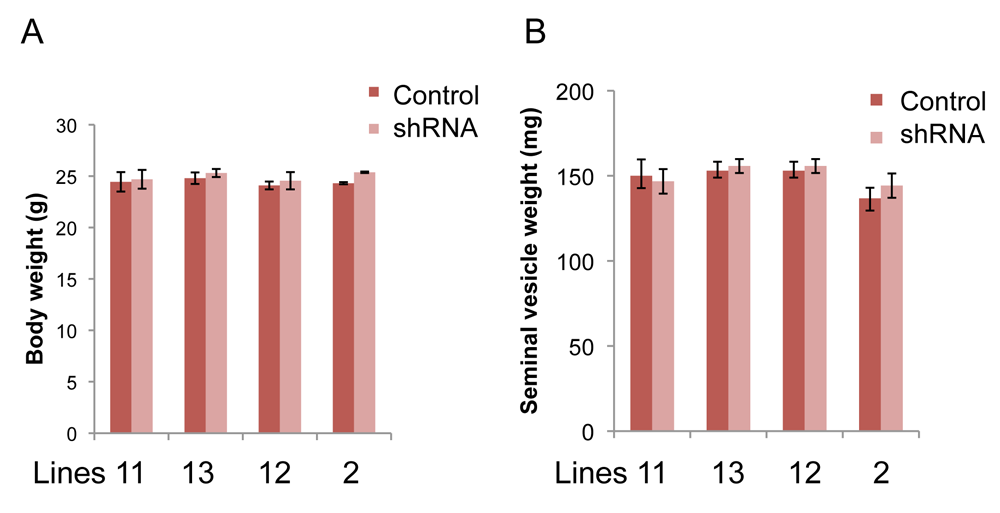

Supplement: S2 Fig — (A) Body weight of Rhox3-shRNA and control mice at 6 weeks of age. (B) Seminal vesicle weight of the Rhox3-shRNA and control mice lines at 6 weeks of age. Values denote the mean ± S.E. Rhox3-shRNA mice, Rhox3-shRNA;Stra8-iCre double-transgenic mice; Control mice, Rhox3-shRNA single-transgenic mice. (TIF) [file pone.0118549.s002.tif]

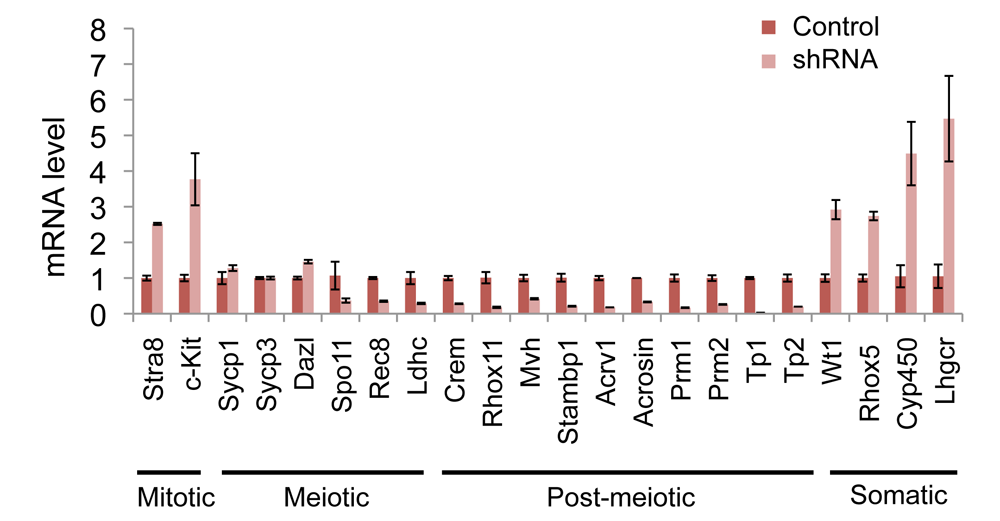

Supplement: S3 Fig — Adult Rhox3-shRNA mice testes have elevated levels of spermatogonial and somatic cell markers and reduced levels of some meiotic and all post-meiotic germ cell markers. qRT-PCR analysis of testes from Rhox3-shRNA and control mice at 6 weeks of age. Values were normalized to the mRNA encoding the ribosomal protein RPL19 and denote the mean ± S.E. Rhox3-shRNA mice, Rhox3-shRNA;Stra8-iCre double-transgenic mice; Control mice, Rhox3-shRNA single-transgenic mice. (TIF) [file pone.0118549.s003.tif]

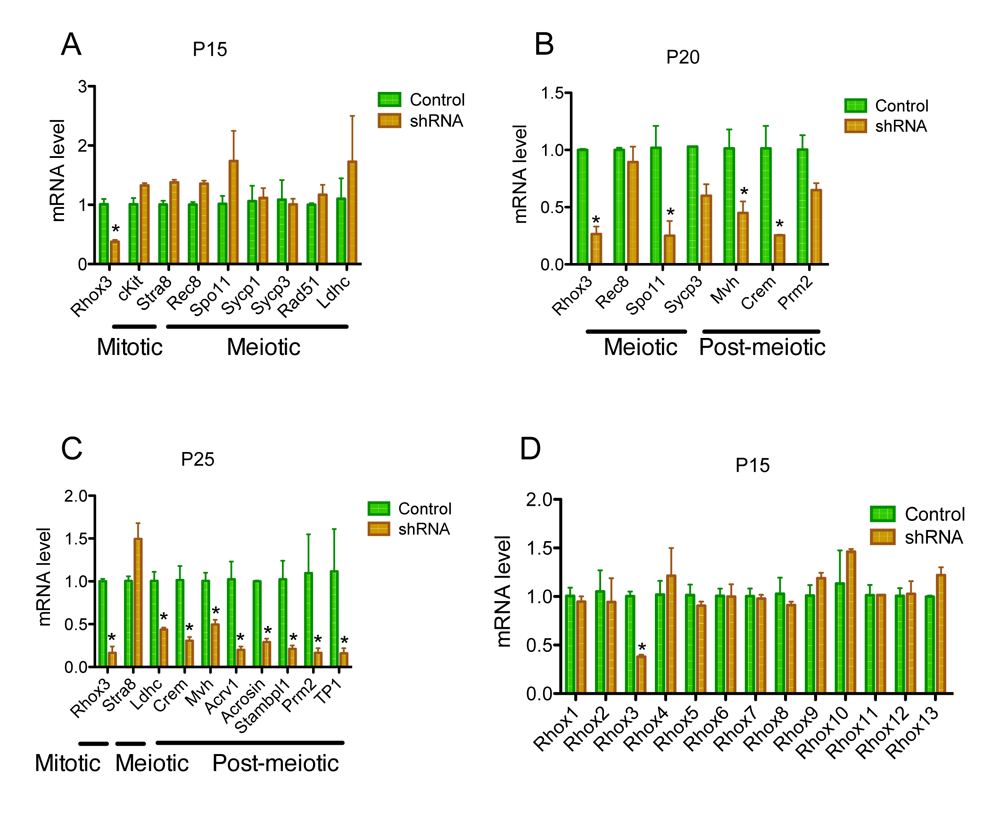

Supplement: S4 Fig — (A–D) qRT-PCR of testes from Rhox3-shRNA and control mice from the indicated postnatal ages. Values were normalized to the mRNA encoding the ribosomal protein RPL19 and denote the mean ± S.E. Asterisk (*) indicates significant changes, p<0.05. Rhox3-shRNA mice, Rhox3-shRNA;Stra8-iCre double-transgenic mice; Control mice, Rhox3-shRNA single-transgenic mice. (TIF) [file pone.0118549.s004.tif]

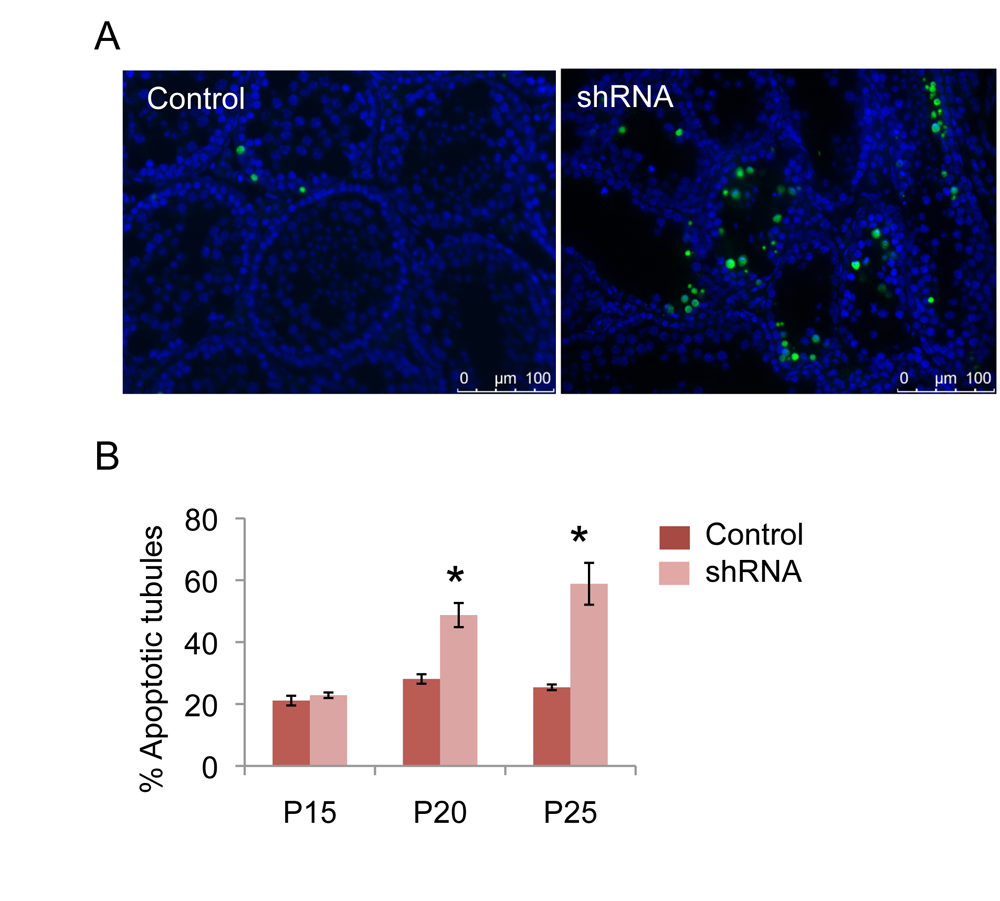

Supplement: S5 Fig — (A) Representative images of TUNEL assay of control and Rhox3-shRNA testes (P25). (B) Quantification of the percentage of seminiferous tubules containing TUNEL-positive cells at the indicated developmental stages. Asterisk (*) indicates significant changes, p<0.05. Rhox3-shRNA mice, Rhox3-shRNA;Stra8-iCre double-transgenic mice; Control mice, Rhox3-shRNA single-transgenic mice. (TIF) [file pone.0118549.s005.tif]
