# Supplementary material for: shRNA Off-Target Effects In Vivo: Impaired Endogenous siRNA Expression and Spermatogenic Defects
Source: PLoS One. 2015 Mar 19;10(3):e0118549. doi: 10.1371/journal.pone.0118549 (PMC4366048; doi:10.1371/journal.pone.0118549)
Supplement: S1 Table — (DOCX) [file pone.0118549.s006.docx]

**Table S1. Fertility of *Rhox3*-shRNA Male Mice.**

| Genotype | n | Litters | Pups | Pups/litter |
| --- | --- | --- | --- | --- |
| Control male x Wild-type female | 4 | 24 | 147 | 6.79 ± 0.44 |
| *Rhox3*-shRNA male x Wild-type female | 4 | 0 | 0 | 0 |

*Rhox3*-shRNA mice, *Rhox3*-shRNA;*Stra8*-iCre double-transgenic mice (Line 2); Control mice, *Rhox3*-shRNA single-transgenic mice.
