# Supplementary material for: shRNA Off-Target Effects In Vivo: Impaired Endogenous siRNA Expression and Spermatogenic Defects
Source: PLoS One. 2015 Mar 19;10(3):e0118549. doi: 10.1371/journal.pone.0118549 (PMC4366048; doi:10.1371/journal.pone.0118549)
Supplement: S2 Table — (DOCX) [file pone.0118549.s007.docx]

**Table S2. Histological Analysis of *Rhox3*-shRNA Testes.**

|  | Cell counts in stages V-early VII tubules | | | Stage I-VIII tubules with RS | Average # of RS/ St-containing tubule | Most advanced type of RS (step) |
| --- | --- | --- | --- | --- | --- | --- |
|  | PS | SC | PS/SC |  |  |  |
| Control | 1044 ± 61.8 | 512 ± 12.2 | 2.18 ± 0.26 | 59 ± 9.8 % | 16.8 ± 0.70 | 4-6 |
| *Rhox3*-shRNA | 420 ± 36.5 | 517 ± 9.7 | 0.81 ± 0.06 | 25 ± 1.6 % | 6.3 ± 0.27 | 3-4 |

PS, pachytene spermatocytes; SC, Sertoli cells; RS, round spermatids; St, spermatids; *Rhox3*-shRNA mice, *Rhox3*-shRNA;*Stra8*-iCre double-transgenic mice; Control mice, *Rhox3*-shRNA single-transgenic mice.
