# Supplementary material for: shRNA Off-Target Effects In Vivo: Impaired Endogenous siRNA Expression and Spermatogenic Defects
Source: PLoS One. 2015 Mar 19;10(3):e0118549. doi: 10.1371/journal.pone.0118549 (PMC4366048; doi:10.1371/journal.pone.0118549)
Supplement: S3 Table — (DOCX) [file pone.0118549.s008.docx]

**Table S3. Microarray Analysis of *Rhox3* shRNA-Regulated Genes.**

| **Gene Symbol** | **RefSeq** | **p-value** | **Fold-Change** |
| --- | --- | --- | --- |
| Pou2af1 | NM_011136 | 0.0198287 | 2.01907 |
| C6 | NM_016704 | 0.043906 | 1.9043 |
| 2310002J15Rik | NM_026415 | 0.0158817 | 1.83982 |
| Rnase9 | NM_183032 | 0.0477059 | 1.82248 |
| Gm5069 | NR_003623 | 0.0235867 | 1.77418 |
| Asb12 | NM_080858 | 0.0203461 | 1.76436 |
| Trem1 | NM_021406 | 0.0486928 | 1.72892 |
| Zpld1 | NM_178720 | 0.0073227 | 1.65433 |
| Pth2 | NM_053256 | 0.0483445 | 1.64529 |
| Prrg4 | NM_178695 | 0.00375609 | 1.63677 |
| Il1b | NM_008361 | 0.0038925 | 1.63518 |
| Clca6 | NM_207208 | 0.0349124 | 1.62915 |
| P2ry14 | NM_133200 | 0.000682903 | 1.62809 |
| Il18r1 | NM_008365 | 0.0168142 | 1.6256 |
| Prg4 | NM_021400 | 0.0166086 | 1.61422 |
| Tmem54 | NM_025452 | 0.012352 | 1.61325 |
| Chi3l1 | NM_007695 | 0.0365986 | 1.60483 |
| Arr3 | NM_133205 | 0.00620713 | 1.60341 |
| Gabra2 | NM_008066 | 0.00375922 | 1.59807 |
| Cxcl1 | NM_008176 | 0.00904788 | 1.58228 |
| Cd80 | NM_009855 | 0.00501213 | 1.5693 |
| Cd19 | NM_009844 | 0.00933275 | 1.56425 |
| Sox11 | NM_009234 | 0.0378992 | 1.55746 |
| Adm2 | NM_182928 | 0.0332391 | 1.55442 |
| Figf | NM_010216 | 0.0434144 | 1.55409 |
| C330046G03Rik | ENSMUST00000092366 | 0.0388024 | 1.53846 |
| Eras | NM_181548 | 0.0164387 | 1.53362 |
| Kcne1 | NM_008424 | 0.00948907 | 1.52375 |
| Krtap26-1 | NM_027105 | 0.0271315 | 1.51198 |
| Vmn1r6 | NM_134175 | 0.00803437 | 1.51129 |
| Cnpy1 | NM_175651 | 0.0352238 | 1.50839 |
| Sh3d20 | NM_183288 | 0.00979138 | 1.50605 |
| Rhd | NM_011270 | 0.00660059 | 1.5031 |
| Cacng5 | NM_080644 | 0.00921236 | 1.50276 |
| Arsa | NM_009713 | 0.020839 | -1.50077 |
| Mgll | NM_001166251 | 0.0342702 | -1.58611 |
| Slc38a5 | NM_172479 | 0.0313491 | -1.61329 |
| Dcdc2a | NM_177577 | 0.0290104 | -1.6762 |
| Tmem160 | NM_026938 | 0.0458238 | -1.70388 |
| Mcrs1 | NM_016766 | 0.00462328 | -1.76033 |
| Cldn2 | NM_016675 | 0.00653027 | -1.85145 |
| Foxf1a | NM_010426 | 0.0220987 | -1.96485 |

All protein-coding genes significantly differentially expressed (P<0.05; >1.5 or <-1.5) between postnatal (P15) *Rhox3-*shRNA and control littermate mice testes (n=3 from each group), as determined by microarray analysis.
